# Resolution of Refractory Chylous Effusions With Targeted MEK Inhibition in NRAS Q61R‐Driven Kaposiform Lymphangiomatosis: A Case Report

**DOI:** 10.1155/crpe/7037459

**Published:** 2026-08-24

**Authors:** Georgia Brown, Sarah McNab, Susan Keogh, John Massie, Lucas Eastaugh, Lydia Pathmanathan, Monique Bertinetti, Michael Sullivan, Lisa Orme, Thomas Cloney, Natasha J. Brown, Tiong Y. Tan, Rebecca Quin, Mark Pertile, Anna Moon, Elhamy Bekhit, Tony Penington

**Affiliations:** ^1^ Pediatric Intensive Care Unit, The Royal Children’s Hospital, Melbourne, Victoria, Australia, rch.org.au; ^2^ Murdoch Children’s Research Institute, Melbourne, Victoria, Australia, mcri.edu.au; ^3^ The University of Melbourne, Melbourne, Victoria, Australia, unimelb.edu.au; ^4^ The Department of General Medicine, The Royal Children’s Hospital, Melbourne, Victoria, Australia, rch.org.au; ^5^ The Department of Respiratory and Sleep Medicine, The Royal Children’s Hospital, Melbourne, Victoria, Australia, rch.org.au; ^6^ The Department of Cardiology, The Royal Children’s Hospital, Melbourne, Victoria, Australia, rch.org.au; ^7^ Vascular Anomalies, The Royal Children’s Hospital, Melbourne, Victoria, Australia, rch.org.au; ^8^ The Department of General Surgery, The Royal Children’s Hospital, Melbourne, Victoria, Australia, rch.org.au; ^9^ The Children’s Cancer Centre, The Royal Children’s Hospital, Melbourne, Victoria, Australia, rch.org.au; ^10^ Victorian Clinical Genetics Service, Melbourne, Victoria, Australia; ^11^ Medical Imaging Department, The Royal Children’s Hospital, Melbourne, Victoria, Australia, rch.org.au

**Keywords:** case report, chylothorax, kaposiform lymphangiomatosis, MEK inhibitor, NRAS mutation, trametinib

## Abstract

**Introduction:**

Kaposiform lymphangiomatosis (KLA) is a rare and aggressive lymphatic anomaly characterised by chylous effusions, respiratory failure and poor prognosis. Diagnosis is often challenging and delayed due to the mosaic distribution of causative somatic mutations.

**Case Presentation:**

A previously well four‐year‐old girl presented with pleural and pericardial effusions and methicillin‐sensitive *Staphylococcus aureus* bacteraemia. She was treated with intravenous antibiotics, and chest and pericardial drains were inserted. Despite these measures, she developed high‐volume, refractory chylous effusions. Management included dietary modification, as well as trials of octreotide and intravenous methylprednisolone, neither of which reduced chyle output. Magnetic resonance lymphangiography demonstrated diffuse abdominal and thoracic lymphatic abnormalities. She was subsequently treated with sirolimus, which was associated with reduced pleural and pericardial chylous drainage but ongoing clinical deterioration. Cell‐free DNA analysis of pleural fluid identified a somatic NRAS variant (NM_002524.5 (NRAS):c.182A > G (p.Gln61Arg)). In the absence of histopathology, a presumed diagnosis of NRAS Q61R‐driven KLA was made based on clinical, radiologic and molecular findings. She was started on trametinib, a MEK inhibitor. Over the subsequent 7 weeks, she was weaned from respiratory support and parenteral nutrition, transitioned to a fat‐containing diet and experienced resolution of clinically significant effusions, allowing discharge home.

**Conclusion:**

This case highlights the diagnostic challenges of complex lymphatic anomalies, demonstrates the utility of cell‐free DNA analysis from effusions in identifying pathogenic somatic variants and underscores the therapeutic potential of targeted MEK inhibition in NRAS Q61R‐driven KLA.

## 1. Introduction

Kaposiform lymphangiomatosis (KLA) is a rare, systemic lymphatic anomaly associated with multifocal lymphatic proliferation and chylous effusions [1]. Children with KLA often present with progressive respiratory compromise, coagulopathy and multisystem involvement. Recent advances in genomic technologies have led to the identification of somatic mutations in the Ras/MAPK signalling pathway, particularly *NRAS* [2]. Standard treatments including dietary modification, medications, immunosuppression and interventional drainage are rarely curative [3]. Recent advances in molecular diagnostics and targeted therapy have opened new therapeutic avenues. This report describes a critically ill child with genetically confirmed KLA and refractory chylous effusions who responded dramatically to targeted MEK inhibition with trametinib.

## 2. Case Presentation

A previously well four‐year‐old girl presented with a 3‐day history of fever, increased work of breathing and cyanosis. Her history included a small muscular ventricular septal defect (VSD), croup and right lobar pneumonia. There was no significant family or medication history.

At a regional hospital, she was febrile and hypoxic. Chest imaging demonstrated a large right‐sided pleural effusion, and intercostal catheter insertion drained purulent fluid. Blood and pleural cultures grew methicillin‐sensitive *Staphylococcus aureus* (MSSA). Intravenous cephazolin and clindamycin were started. Echocardiography revealed a large pericardial effusion, prompting transfer to a tertiary paediatric centre for pericardiocentesis and ongoing management.

**FIGURE 1:**
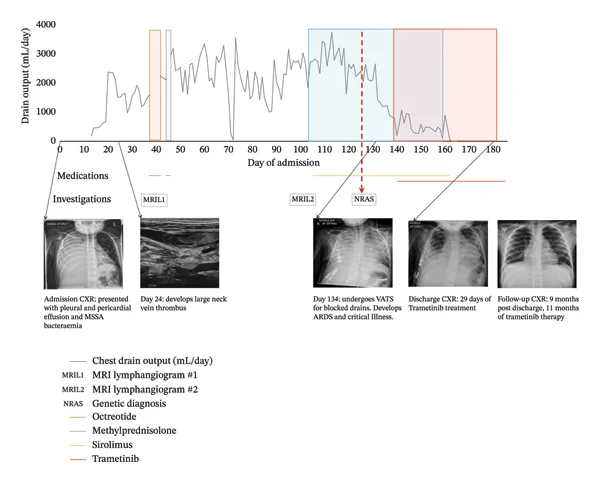
Clinical timeline.

Both pleural and pericardial fluid were yellow and cloudy with elevated triglycerides and a lymphocytic predominance, consistent with chylous effusions. Standard therapies—including low‐fat diet, parenteral nutrition, corticosteroids and octreotide—failed to reduce drain output (Figure 1). The patient demonstrated thrombocytosis and microcytic anaemia with normal coagulation parameters.

A peripherally inserted central catheter was placed in the left arm for antibiotics and parenteral nutrition. Subsequently, she developed an extensive occlusive thrombus involving the left subclavian and internal jugular veins, extending into the brachiocephalic vein, which was associated with increased chylous drainage. Therapeutic anticoagulation with heparin was initiated.

### 2.1. Diagnostic Assessment

**FIGURE 2:**
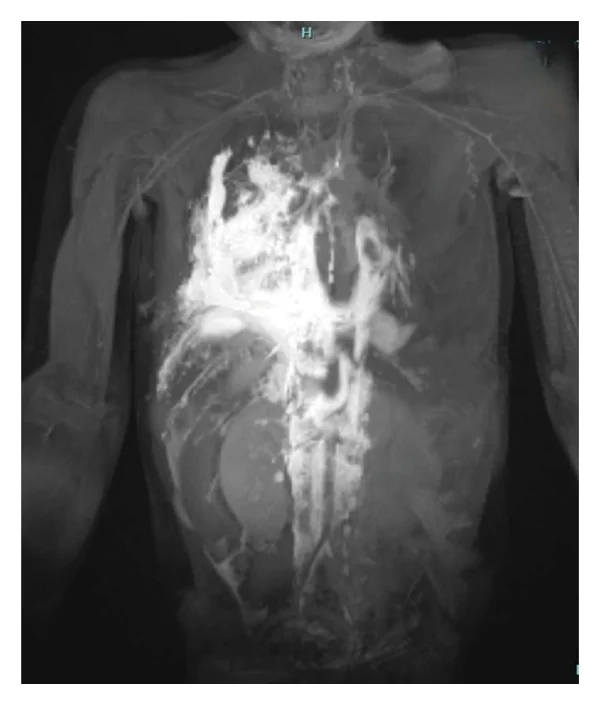
Coronal T1‐weighted MR lymphangiogram following intrahepatic lymphatic contrast injection, demonstrating abnormal dilated lymphatic channels within the mediastinum and upper abdomen, with associated right pleural and pericardial effusions.

Femoral intranodal magnetic resonance lymphangiography was technically challenging and nondiagnostic for delineating a specific lymphatic anomaly or thoracic duct anatomy or patency. Repeat MR imaging via hepatic, mesenteric and inguinal nodes revealed extensive abnormalities suggestive of a generalised lymphatic anomaly (GLE) (Figure 2).

Cardiac catheterisation was undertaken to exclude secondary causes of chylothorax such as significant VSD shunting or diastolic dysfunction. The study demonstrated no significant VSD shunt, normal diastolic pressures and normal pulmonary pressures with estimated vascular resistance within the normal range.

Single nucleotide polymorphism (SNP) microarray on blood derived DNA was normal. DNA extracted from lymphocyte depleted pleural fluid samples was run on a next generation sequencing Somatic Tumour Gene Panel comprising 26 genes, including *NRAS,* with coverage of approximately 500X. The results were negative. Following international consultation, a pleural fluid sample was sent to the Children’s Hospital of Philadelphia for cell‐free DNA (cfDNA) testing. Pleural fluid was collected in Streck tubes (Streck Cell‐Free DNA BCT tubes (Streck, La Vista), spun and supernatant was transferred to LoBind tubes (Eppendorf DNA LoBind Tubes [Eppendorf, Hamburg, Germany]). Samples were shipped on ice. Using a targeted 51 gene panel for vascular malformations, a recurrent somatic *NRAS* variant was identified: NM_002524.5 (NRAS):c.182A > G (p.Gln61Arg); variant allele fraction was 4.69%.

Prior to molecular diagnosis, the diffuse lymphatic abnormalities demonstrated on lymphangiography were considered most consistent with a GLA. Following identification of the pathogenic NRAS p.Gln61Arg (Q61R) variant in pleural fluid cfDNA, the diagnosis was revised to presumed NRAS Q61R‐driven KLA, reflecting the established association between this variant and KLA. This molecular diagnosis informed the decision to pursue targeted MEK inhibition with trametinib.

**FIGURE 3:**
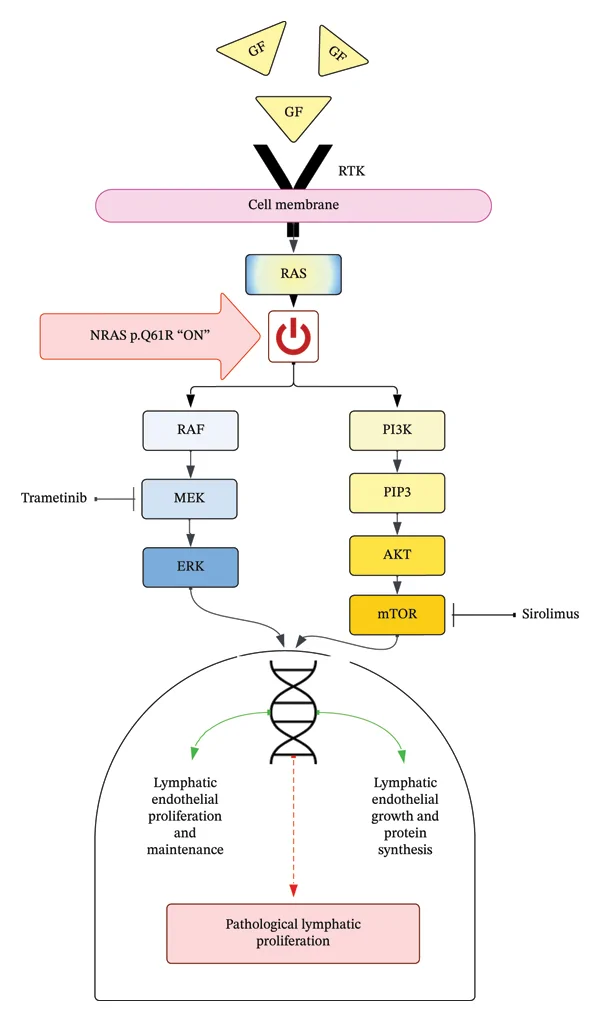
Schematic representation of the RAS/MAPK and PI3K/AKT/mTOR signalling pathways. Growth factors (GF) activate receptor tyrosine kinases (RTK) at the cell membrane, leading to downstream signalling through NRAS. In normal physiology (green), these cascades regulate cell growth, metabolism and turnover. In this case, a pathogenic NRAS variant drives constitutive (red) activation, keeping the pathways “switched on.” MEK inhibition (trametinib) and mTOR inhibition (sirolimus) are shown at their respective sites of action. Abbreviations: GF, growth factor; RTK, receptor tyrosine kinase; NRAS, neuroblastoma RAS viral oncogene homologue; MAPK, mitogen‐activated protein kinase; MEK, MAPK/ERK kinase; ERK, extracellular signal‐regulated kinase; PI3K, phosphoinositide‐3‐kinase; AKT, protein kinase B; mTOR, mechanistic target of rapamycin.

Access to trametinib was obtained through a compassionate access program, as the drug is not approved or PBS‐listed in Australia for paediatric lymphatic anomalies. Approval was granted after submission to the manufacturer and institutional governance, permitting off‐label use in this child with NRAS Q61R‐driven KLA. Whilst awaiting approval and given the potential crosstalk between the RAS/MAPK and PI3K/AKT/mTOR pathways (Figure 3), sirolimus was trialled (initiated at 1.12 mg/m^2^/day, targeting trough level 5–10 μg/L). Over 8 weeks of sirolimus treatment, there was a reduction in pleural and pericardial drain output; however, therapy was discontinued due to clinical deterioration and the development of pancreatitis, a recognised complication of sirolimus. Trametinib was subsequently introduced, while sirolimus was being weaned, resulting in approximately 3 weeks of overlapping therapy before sirolimus was discontinued (Figure 1)

On day 137 of admission, trametinib treatment was initiated at 0.5 mg enterally once daily. Shortly thereafter, the patient deteriorated with blocked drains, recurrent effusions and cardiac tamponade. She required emergent video‐assisted thoracoscopic surgery (VATS) with insertion of new chest and pericardial drains. Postoperatively, she developed profound hypoxaemic respiratory and multiorgan failure, necessitating intensive care admission, escalation to high‐frequency oscillatory ventilation and consideration of extracorporeal membrane oxygenation (ECMO). The family was counselled regarding her poor prognosis, and chest drains were removed at their request to allow them to hold her in what were expected to be her final moments. Unexpectedly, following drain removal and lateral positioning, oxygenation improved and stabilised.

Over the subsequent days, she was weaned from mechanical ventilation and supplemental oxygen. Continued trametinib therapy was associated with progressive improvement in respiratory status and sustained resolution of chylous effusions. Other medications were discontinued, parenteral nutrition was ceased and a fat‐containing diet was successfully reintroduced. Seven weeks after initiation of trametinib, she was discharged home on trametinib 0.5 mg daily after a total hospital stay of 186 days.

### 2.2. Follow‐Up and Outcomes

At 3 months postdischarge, the patient remained clinically well with only a small residual pleural effusion. By 6 months, she had returned to school and remained asymptomatic while continuing trametinib therapy. At 14 months, she demonstrated normal growth and activity with minimal treatment toxicity, limited to mild skin dryness and a low‐grade follicular rash. She continues to demonstrate sustained clinical and radiologic response.

## 3. Discussion

KLA is recognised within the group of complicated lymphatic anomalies and is characterised by aggressive and progressive disease [1–4]. Clinically, patients typically present in childhood with recurrent or refractory chylous effusions, respiratory compromise, and in some cases, consumptive coagulopathy or bleeding diathesis. Other features may include pericardial effusion, ascites, bony involvement and splenic or mediastinal disease.

Diagnosis is challenging and often delayed, not only because of the mosaic distribution of underlying somatic variants but also due to the rarity of the condition and limited clinician familiarity, which can lead to misdiagnosis or prolonged empiric management. Recommended evaluation includes cross‐sectional imaging to define disease extent, dynamic contrast MR lymphangiography to assess thoracic duct and abnormal drainage and laboratory assessment of haematologic and coagulation parameters [3, 4]. Angiopoietin‐2 may serve as a biomarker of disease activity, and genetic testing of lesional tissue or cell‐free DNA can identify pathogenic variants such as NRAS Q61R [5]. Histopathology, when available, demonstrates malformed lymphatic channels accompanied by clusters or sheets of spindle‐shaped (‘kaposiform’) lymphatic endothelial cells, although biopsy is often avoided due to procedural risk [1, 2, 4, 6]. These combined clinical, radiologic, laboratory and molecular features form the basis for diagnosis and guide targeted therapeutic approaches.

The negative SNP microarray and somatic tumour panel highlight the limitations of standard clinical testing in detecting low‐level mosaic variants. In recent years, with improvements in capabilities for detecting low level mosaic variants, molecular testing has become central to diagnosis. However, these new methods take time to be incorporated into clinical care and access to new diagnostic methods is often restricted initially to research programs.

Recurrent somatic gain of function variants in *NRAS*, particularly c.182A > G (p.Gln61Arg), have been identified as disease drivers in many cases of KLA [2, 7]. These variants may be present at such low variant fractions that they are not detectable on standard clinical gene panels, where coverage typically is ∼500–1000X, as in this case. The variants are more likely to be identified within DNA from endothelial cells, therefore the handling and separation of pleural fluid samples, which contain a wide range of cell types, is critical to establishing a diagnosis [8]. These low‐level variants may be detectable through analysis of cfDNA obtained from effusion fluid [8]. The ability to establish a diagnosis using effusion cfDNA can be technically challenging but is especially valuable in critically ill patients where surgical biopsy may be high‐risk or infeasible.

In our case, molecular confirmation via cfDNA analysis played a decisive role in both diagnosis and treatment planning. The patient’s initial presentation mimicked complicated bacterial empyema with pericardial involvement. However, the persistence of high‐volume effusions and failure to respond to antimicrobial therapy and conventional supportive measures prompted a broader diagnostic evaluation. MR lymphangiography demonstrated diffuse lymphatic abnormalities initially considered most consistent with a GLA. Subsequent cfDNA analysis of pleural fluid identified a pathogenic NRAS p.Gln61Arg (Q61R) variant, leading to a revision of the diagnosis to presumed NRAS Q61R‐driven KLA. This molecular diagnosis was pivotal, as it directly informed the decision to pursue targeted MEK inhibition with trametinib.

While the clinical presentation was consistent with KLA, the diagnosis remains presumptive in this case. The patient did not exhibit thrombocytopenia, skeletal lesions or a discrete enhancing mediastinal soft tissue mass—features classically associated with KLA [2–4]. Histopathological confirmation was not feasible due to the location of the lymphatic abnormalities and patient’s clinical state. Nonetheless, the constellation of chylous effusions, diffuse thoracic lymphatic abnormality on lymphangiography and detection of a recurrent *NRAS* variant supported the clinical diagnosis. While *NRAS* mutations are strongly associated with KLA, they are not currently considered pathognomonic [4]. We, therefore, refer to this as a presumed diagnosis of *NRAS Q61R-driven* KLA, consistent with current diagnostic approaches in critically ill patients where biopsy is not feasible [3, 4].

Somatic NRAS variants, most commonly affecting Codon 61 (e.g., p.Gln61Arg), result in constitutive activation of the RAS/MAPK signalling cascade, driving abnormal lymphatic endothelial cell proliferation and survival. Cross‐talk between the RAS/MAPK and PI3K/AKT/mTOR pathways may also contribute to disease phenotype, although MEK inhibition directly targets the aberrant RAS‐driven signalling that underlies KLA [5].

In this case, sirolimus was associated with some reduction in chylous drainage, indicating biological activity even when the primary pathogenic driver is within the RAS/MAPK pathway [7]. An important consideration is that there was a period of approximately 3 weeks during which sirolimus and trametinib were administered concurrently. Consequently, additive or synergistic therapeutic effect cannot be excluded. However, although sirolimus was associated with reduced drain output, it did not result in clinical resolution of disease, and the patient continued to deteriorate clinically. In contrast, sustained clinical recovery, resolution of effusions and eventual hospital discharge occurred following initiation and continuation of trametinib.

Identification of an NRAS mutation subsequently enabled targeted therapy with trametinib, which, although associated with a delayed onset of clinical improvement of around 4 weeks, led to durable disease control and recovery. This highlights the value of incorporating genetic testing into the diagnostic pathway for complex lymphatic anomalies, both to avoid unnecessary morbidity from less effective therapies and to enable early access to pathway‐specific treatments.

This case highlights the critical importance of timely access to advanced molecular diagnostics and cross‐disciplinary collaboration. In a child with a prolonged and severe course, the integration of research based cfDNA testing enabled precision therapy and fundamentally altered the clinical trajectory. While previous reports have documented trametinib efficacy in similar contexts, the completeness of this patient’s recovery was particularly striking, especially given her initial severity of illness and poor prognosis [9].

Beyond the diagnostic and therapeutic success, this case highlights several key principles in managing complex lymphatic anomalies. High‐output chylous effusions can lead to significant metabolic derangements, micronutrient deficiencies and immunosuppression, requiring sustained multidisciplinary input for stabilisation. Thrombotic complications—observed in this case as a central venous thrombosis, can further impair lymphatic return and exacerbate lymphatic leakage. Additionally, invasive procedures may trigger inflammatory cascades or worsen lymphatic leakage and should be approached cautiously in this population.

## Funding

Open access publishing was facilitated by The University of Melbourne, as part of the Wiley–The University of Melbourne agreement via the Council of Australasian University Librarians.

## Ethics Statement

Ethical review was not required for this single‐patient case report in accordance with the local institutional guidelines. Written informed consent was obtained from the patient’s legal guardian, and institutional support for publication was obtained from the Head of Department.

## Conflicts of Interest

The authors declare no conflicts of interest.

## Patient Perspective

The family hopes that sharing their experience will contribute to greater understanding and help accelerate access to timely and effective care and therapies for other children living with similar conditions.

## Data Availability

The data that support the findings of this study are available from the corresponding author upon reasonable request.
